# Evolved DNAzymes and Stable Activation Chemistry Enable High‐Efficiency DNA Ligation

**DOI:** 10.1002/chem.202502788

**Published:** 2025-12-05

**Authors:** Connor Nurmi, Gemma Mendonsa, Mengdi Bao, Yingfu Li

**Affiliations:** ^1^ Department of Biochemistry and Biomedical Sciences, McMaster University Hamilton Ontario Canada; ^2^ Biointerfaces Institute McMaster University Hamilton Ontario Canada; ^3^ Seagate Research Group Seagate Technology Shakopee USA

**Keywords:** Activation chemistry, DNA data storage, DNA Ligation, DNAzyme, In vitro selection

## Abstract

DNA ligation is a fundamental reaction used in a variety of applications, typically performed by T4 DNA ligase. For certain applications, such as DNA data storage, DNA‐ligating DNAzymes offer a more stable and cost‐effective alternative to protein enzymes. The E47 DNAzyme is among the most efficient DNA‐ligating DNAzymes reported, but it still lacks sufficient activity for widespread adoption and requires a highly unstable phosphoimidazole‐activated DNA substrate to function. In this study, we performed in vitro selection using a pre‐structured library based on E47 and identified sequences with more than a twofold increase in ligation activity, representing the fastest DNA‐ligating DNAzymes reported to date. We also screened alternative imidazolide compounds for substrate activation and found that phosphobenzimidazole‐activated DNA substrates are significantly more stable, remaining intact for at least 24 h at room temperature. These advances improve the practicality of DNAzymes for ligation‐based applications and broaden their potential use in DNA data storage.

## Graphical abstract

In vitro selection of pre‐structured DNA libraries and chemical screening of alternative activation groups yielded *trans*‐acting DNAzymes with faster ligation rates and enhanced DNA substrate stability. The combination of C3S1 DNAzymes and phosphobenzimidazole‐activated DNA substrates provides improved DNA ligation performance, offering promise for applications such as DNA data storage.

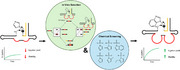

## 1 Introduction

Enzymatic DNA ligation is vital for many cellular processes such as recombination, replication, and repair of DNA [1], and can also be applied to a variety of biotechnological applications, including DNA cloning [2, 3], next‐generation DNA sequencing [4], nucleic acid detection [5, 6, 7], and DNA computation [8, 9]. T4 DNA ligase, which catalyzes the ATP‐dependent formation of a phosphodiester bond between adjacent 5′‐phosphate and 3′‐hydroxyl moieties in DNA, is often employed for these applications [10]. However, in select cases, such as DNA data storage, rapid, protein‐free, and sequence‐specific DNA ligation is needed. Catalytic DNA molecules (DNAzymes) are an attractive alternative, primarily due to their lower production cost and higher long‐term stability. Novel digital DNA data storage methods have used DNAzymes, instead of T4 DNA ligase, for rapid parallel ligation of data‐encoded sequences because high reaction volumes substantially increase the cost of protein‐based enzymes [11]. DNA‐ligating DNAzymes are also advantageous because they offer longer shelf‐life compared to T4 DNA ligase [12, 13], which is critical for eventual integration within commercial data storage units.

DNA‐ligating DNAzymes are developed via in vitro selection [14, 15, 16] and use Watson‐Crick (WC) duplex formation to ligate almost any two single‐stranded DNA (ssDNA) molecules, provided the two duplex binding arms are designed with complementary sequences to the two ssDNA substrates. Compared to other DNAzyme classes, such as RNA‐cleaving DNAzymes, there is a relatively limited number of DNAzymes with ligation activity. Each reported DNA‐ligating DNAzyme is summarized in Table S1, outlining their varying degrees of performance and utility.

One of the earliest DNA‐ligating DNAzymes, C1, possessed a relatively high rate of DNA ligation with a first‐order rate constant (*k*_obs_) of 0.033 min^−1^. However, it required chemically modified 3′‐sulfur and 5′‐iodine substrate nucleotides, forming a nonconventional phosphorothioate linkage, which is likely to inhibit downstream reactions and increase overall reaction cost [17]. Additionally, C1 was only active in the *cis* conformation, where the DNA substrate is ligated to the DNAzyme, forming a single product oligonucleotide consisting of the combined DNA substrate and DNAzyme. By contrast, *trans*‐acting DNA‐ligating DNAzymes ligate two separate DNA substrates without incorporating the DNAzyme into the ligation product and are thus more desirable for practical applications.

An early *trans*‐acting DNA‐ligating DNAzyme, L115, catalyzed the sequence‐specific ligation of an adenylated DNA substrate with a *k*_obs_ of 0.001 min^−1^‐33‐fold slower than C1 [18]. Although L115 was initially selected as a *cis*‐acting DNAzyme, it was later converted into a functional *trans*‐acting DNAzyme. However, the strict sequence requirements of the adenylated DNA substrate and the 3′ terminus of L115 restricted its substrate versatility, reducing overall utility. The fastest DNA‐ligating DNAzyme reported is 8LV13, which catalyzed ligation with an adenylated DNA substrate in *trans* with a *k*_obs_ of 0.1 min^−1^. However, its utility is limited as it generates only non‐canonical 2′‐5′ linkages for branched DNA polymers [19].

The E47 DNAzyme was the first DNA‐ligating DNAzyme reported and arguably has the greatest overall utility and performance among this class. It possesses a modest *k*_obs_ of 0.056 min^−1^ in *trans*, with modifiable 5′ and 3′ substrate recognition arms that increase the versatility of sequences to be ligated [20]. Recently, Mendonsa and colleagues demonstrated the high versatility of E47 substrate recognition arm design by ligating five unique data‐encoded sequences in parallel [11]. E47 also forms a canonical 3′‐5′ phosphodiester linkage, without the need for nucleic acid modification, enabling the ligation products to be used as substrates in downstream reactions, such as next‐generation sequencing—a critical step in the DNA data storage process [21].

Despite the advantages of E47, two main issues prevent its widespread adoption. First, the rate of DNA ligation needs improvement. For example, in the DNAzyme‐based DNA data storage study mentioned earlier, lower ligation yields with E47 were observed as product length increaseda necessary feature for encoding longer data sequences [11]. DNAzymes with improved ligation activity could therefore enhance the speed at which data‐encoded sequences are ligated in parallel, increasing overall “write” speed. Second, E47 requires a preliminary substrate activation step with imidazole and carbodiimide (EDC) to generate a phosphoimidazole group on the 3′ terminal nucleotide of the DNA substrate to be ligated. This intermediate is highly unstable and degrades rapidly at room temperature [22]. Thus, to improve the overall utility of E47, the rate of DNA ligation must be improved while simultaneously increasing the stability of the activated DNA substrate.

Herein, we describe the in vitro selection of new DNA‐ligating DNAzymes using a pre‐structured library based on E47. After 10 rounds of selection and sequence characterization, we identified a sequence cluster with more than a twofold increase in ligation rate compared to the original *trans*‐acting E47 and investigated the sequence requirements of DNA substrates. Lastly, we explored alternative phosphoimidazole activation chemistries to improve substrate stability and found that phosphobenzimidazole activation was significantly more stable, enabling comparable ligation yields even after 24 h at room temperature.

## 2 Results and Discussion

### 2.1 In Vitro Selection Using a Pre‐Structured Library Based on E47

Our first objective was to obtain DNAzymes with DNA ligase activity via in vitro selection. There were two main strategies that we considered for the initial library design: completely randomized libraries or pre‐structured libraries. The original selection process that identified the E47 DNAzyme employed a completely randomized library of 116 nucleotides, corresponding to 6.9 × 10^69^ unique sequences [20]. However, due to practical limitations, the initial library contained 3.5 × 10^14^ unique sequences, meaning that only an extremely small fraction of the total sequence space was sampled. The benefit of a larger random region is greater structural diversity, albeit at the expense of sequence space coverage [23, 24]. Pre‐structured libraries, by contrast, reduce the number of unique sequences by focusing the in vitro selection on functionally critical nucleotides [25]. In the case of E47, we hypothesized that maintaining the pre‐structured regions of the catalytic core would improve the coverage of sequence space for more functionally important randomized nucleotides elsewhere in the catalytic core. Thus, we chose the pre‐structured library approach for the in vitro selection, retaining the advantages of an all‐DNA system and the ability to recognize the ligating substrates via two DNA duplexes.

**FIGURE 1:**
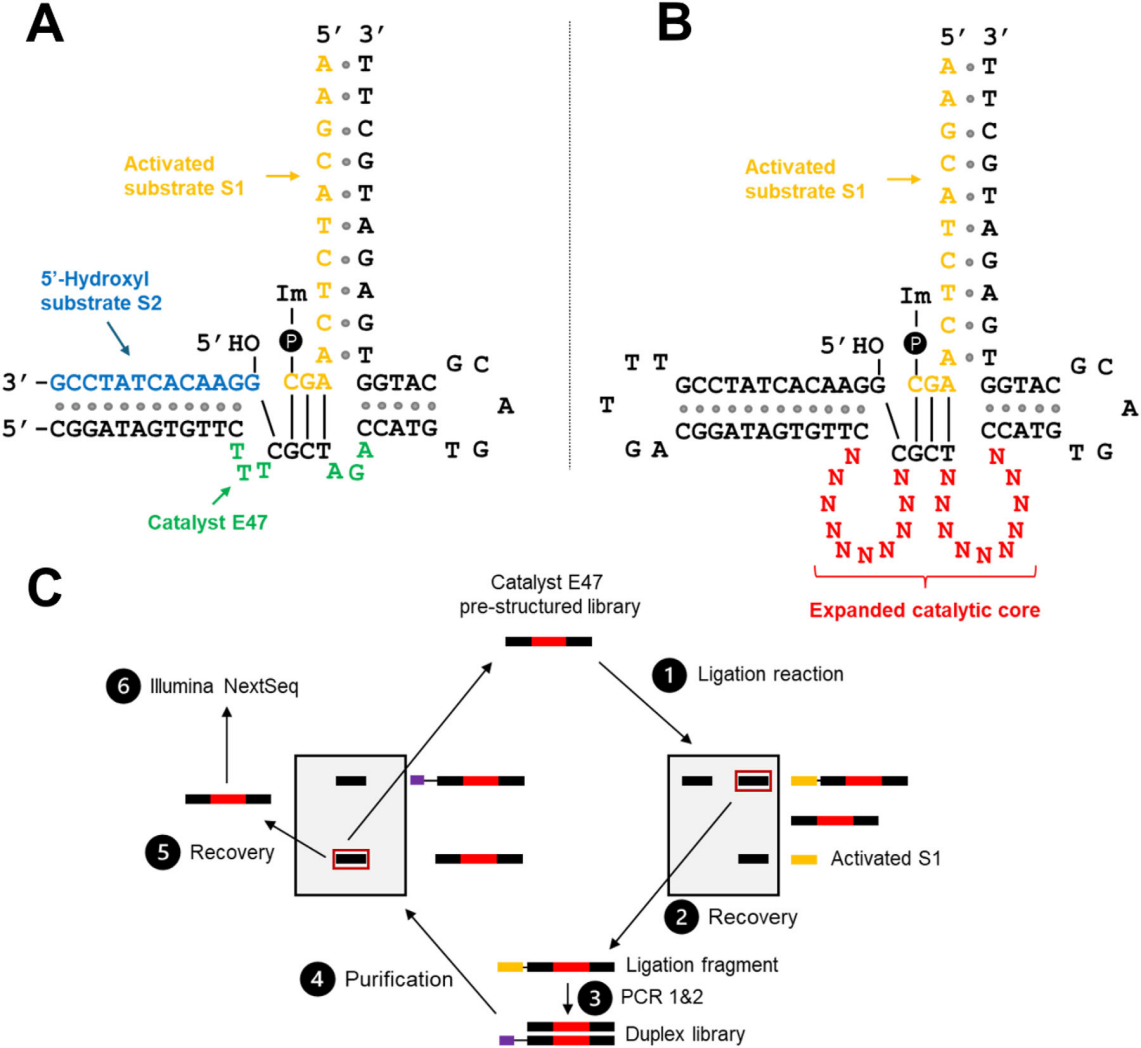
Structure of the original Zn^2+^‐dependent, *trans*‐acting DNA ligase DNAzyme, E47, with the catalytic core sequence in green, Im = Imidazole and 🅟 = Phosphate (A). The EDC‐activated 3’‐phosphoimidazole S1 DNA substrate is yellow, while the S2 DNA substrate is blue for *trans*‐ligating systems. The in vitro selection library is designed in a *cis* conformation and maintains the central “CGCT” motif, flanked by two expanded 10‐nt random nucleotide (N_10_) regions (B). Schematic of the in vitro selection used to isolate DNA sequences with improved ligase activity compared to catalyst E47 (C). 1) The library was incubated with purified, 5′‐^32^P radiolabeled and 3′‐phosphoimidazole‐activated S1 DNA substrate. 2) The ligated product was purified by 10% denaturing PAGE with 8 M urea (dPAGE). 3) The purified products were amplified by PCR. 4) The PCR products were subjected to dPAGE to separate the sense strand from the antisense strand. 5) The sense strand was recovered and used in the next round of in vitro selection. 6) A portion of the recovered sense strand was kept for use for Illumina NextSeq sequencing.

The core of the E47 DNAzyme is relatively short, consisting of only 10 nucleotides (5′‐TTTCGCTAGA‐3′) (Figure 1A). If the catalytic core (N_10_) of E47 is completely randomized, it would only yield ∼ 1 million unique sequences, much less than the typical 10^14^ or 10^15^ unique sequences present in most in vitro selection experiments. Thus, we designed a starting library with a larger random domain of 20 nucleotides (10^12^ unique sequences), while maintaining the structural DNA substrate‐binding properties of the central CGCT motif. We hypothesized that increasing the length of the randomized domain would enable the adoption of a broader range of catalytic structures, thereby increasing the likelihood of folding into a more catalytically active conformation. Specifically, we replaced the catalytic core regions that do not hybridize with the S1 DNA substrate — TTT and AGA — with 10 randomized nucleotides (N_10_) each, resulting in a total randomized region of 20 nucleotides (N_20_) (Figure 1B).

The overall in vitro selection schematic is shown in Figure 1C, where successfully ligated library sequences were isolated, amplified, and then used in the subsequent round of selection. Between in vitro selection rounds 1–4, fewer than 1% of library sequences were successfully ligated, but in round 5, significant enrichment of the pool was observed, where the ligation yield of the pool increased from 0.12% to 7.4% (Figure S1). After 9 rounds of selection, further enrichment in DNA ligase activity was no longer observed, with the ligation yield of the pool reaching 2.2% within 30 s. The final 10^th^ round of selection was performed with reduced library and S1 DNA substrate concentrations (10 pmol instead of 25 pmol), a common strategy to select for sequences with more rapid association rates (*k*_on_) [26]. Before sequencing, we performed a quality control experiment to observe the relative S1 DNA ligation activity of each round and compared it to the DNA ligation activity of the original *cis*‐acting E47. Consistent with our in vitro selection findings, we observed a significant increase in DNA ligation activity between rounds 4 and 5, with 1.7% and 9.4% ligated, respectively (Figure S2). Rounds 8 (16.1%) and 9 (16.3%) were the only pools with higher DNA ligation activity than *cis*‐acting E47 (15.8%), indicating that sequences with faster S1 DNA ligation activity compared to catalyst E47 may have evolved during the in vitro selection process.

All 10 rounds of in vitro selection were sequenced by Illumina NextSeq sequencing and organized into sequence clusters, which contained sequences with no more than 5% nucleotide variation. The top sequence clusters were chosen based on two main parameters: 1) abundance throughout the selection and 2) rate of enrichment (Figure S3). We selected a total of 11 sequences across 6 different clusters. Clusters 1, 3, 4, and 6 were chosen based on their high abundance in rounds 8–10 of in vitro selection, while clusters 2 and 5 were chosen due to their high trajectory of enrichment, showing sequence enrichment of over four orders of magnitude (10^4^) between subsequent rounds.

### 2.2 DNA Ligase Kinetics of Isolated Sequences

**FIGURE 2:**
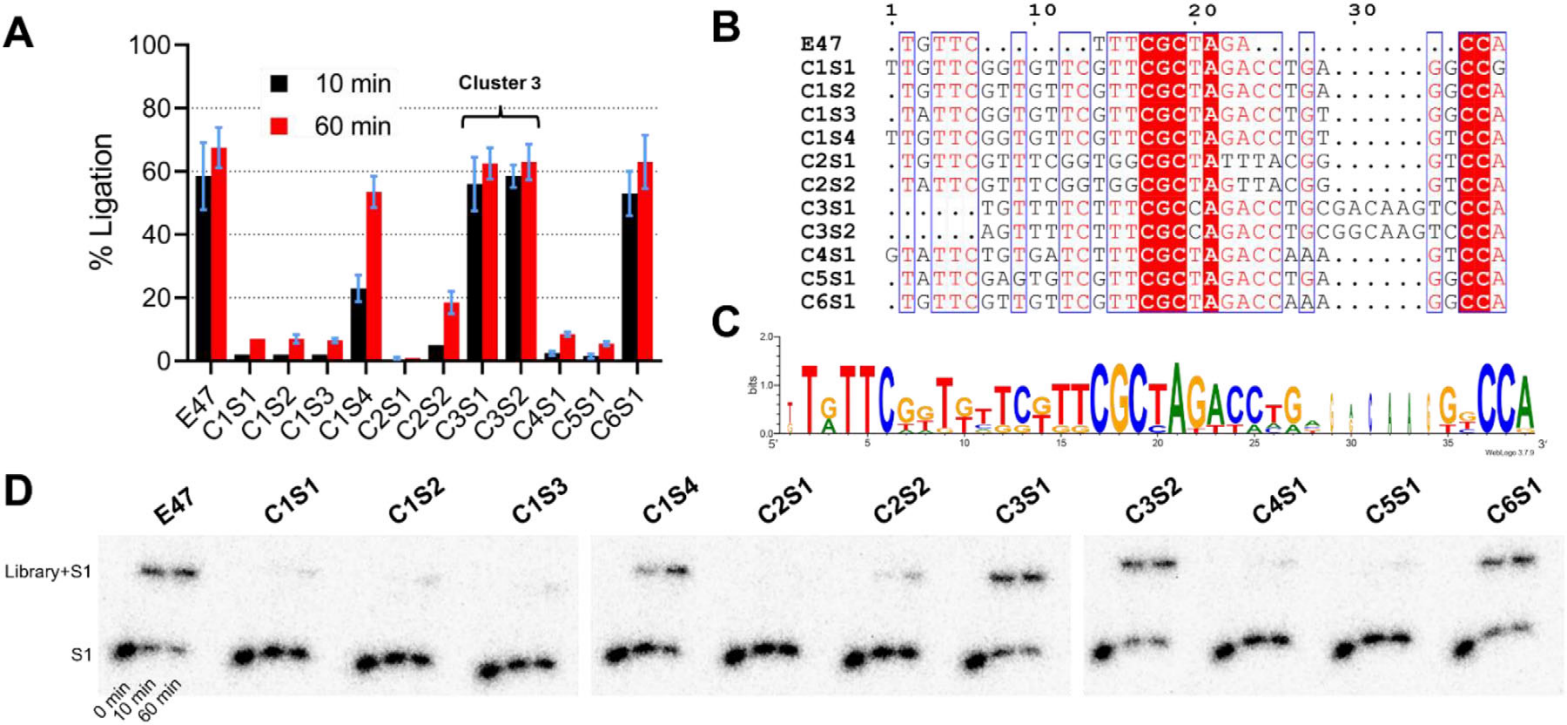
DNA ligation activity of 11 sequences from the top 6 clusters in round 10 of the in vitro selection plus E47, performed in triplicate (A). Sequence alignment of the 11 sequences from the top 6 clusters plus the original E47 DNAzyme was generated using ESPript3, with conserved nucleotides highlighted in red and semi‐conserved nucleotides boxed in blue (B). Sequence logo analysis of the 11 sequences from the top 6 clusters plus E47 is shown, where nucleotide height (bits) corresponds to the likelihood of a nucleotide existing at a given position and width corresponds to prevalence (C). DNA ligation activity was examined by dPAGE, with timepoints taken before addition of reaction buffer containing 4 mM Zn^2+^ (0 min), and after 10 min and 60 min following buffer addition (D).

We performed a DNA ligation activity screen with the 11 sequences (*cis* conformation) isolated from the top 6 clusters (Figure 2A). Four sequences (C1S1‐S4) were chosen from cluster 1, two sequences from cluster 2 (C2S1, C2S2), and cluster 3 (C3S1, C3S2), and one sequence each from clusters 4 (C4S1), 5 (C5S1), and 6 (C6S1). Comparing the DNA ligation activity of each sequence to *cis*‐E47 revealed several highly active and low activity sequences.

Starting with the sequences from cluster 1, which comprised 74% of all sequence clusters in round 10, only C1S4 was active. From the sequence alignment, all four sequences retained the same catalytic core sequence (5′‐TTTCGCTAGA‐3′) as the E47 DNAzyme, except for a T→G substitution at position 14 (Figure 2B, C). Comparing C1S4 to the other three sequences in cluster 1, C1S4 possessed a T→G substitution at position 36, which may have enabled more conformation flexibility by eliminating the short stem in the catalytic core (Figure S4). Nevertheless, C1S4 was still significantly less active compared to E47, indicating that these structural changes did not confer improved ligation ability.

Cluster 2, which comprised 6% of all sequences in round 10, showed changes in the TTT (positions 14–16) and AGA (positions 21–23) motifs flanking the central CGCT motif. Both C2S1 and C2S2 possessed significantly lower activity than E47. C2S1 was completely inactive and was the only sequence among the 11 tested with no detectable ligation bands by dPAGE (Figure 2D). C2S1 had the TTT motif changed to TGG and AGA motif changed to ATT, while C2S2 retained some activity with a change to AGT. For C2S1, the complete loss of activity following the G→T substitution at position 22 highlights its importance in catalysis, since no other sequence contained a substitution at this position.

Cluster 3, which comprised 4% of all sequences in round 10, displayed DNA ligation activity comparable to E47 with both C3S1 and C3S2. Cluster 3 was unique as the only cluster with an altered CGCT motif, which was changed to CGCC. Sequence conservation with this motif showed that the first three CGC nucleotides are absolutely conserved, while T can tolerate substitution with C at position 20. This C‐A mismatch with the S1 substrate did not impair ligation activity, and may even improve it, as explored further in the subsequent section.

For the remaining clusters, only one sequence was tested per cluster. For cluster 4 (C4S1) and cluster 5 (C5S1), which comprised 1% and 0.2% of all sequences in round 10, respectively, limited activity was observed. C4S1 retained the same catalytic core sequence (5′‐TTTCGCTAGA‐3′) as E47, while C5S1 substituted the TTT motif with GTT, as in cluster 1. The limited activity of C4S1 and C5S1 was likely due to sequence differences outside of the catalytic core, which may have inhibited proper folding. Cluster 6 (C6S1) was the exception, possessing the same catalytic core sequences as cluster 5, but with ligation activity comparable to cluster 3 and E47.

**FIGURE 3:**
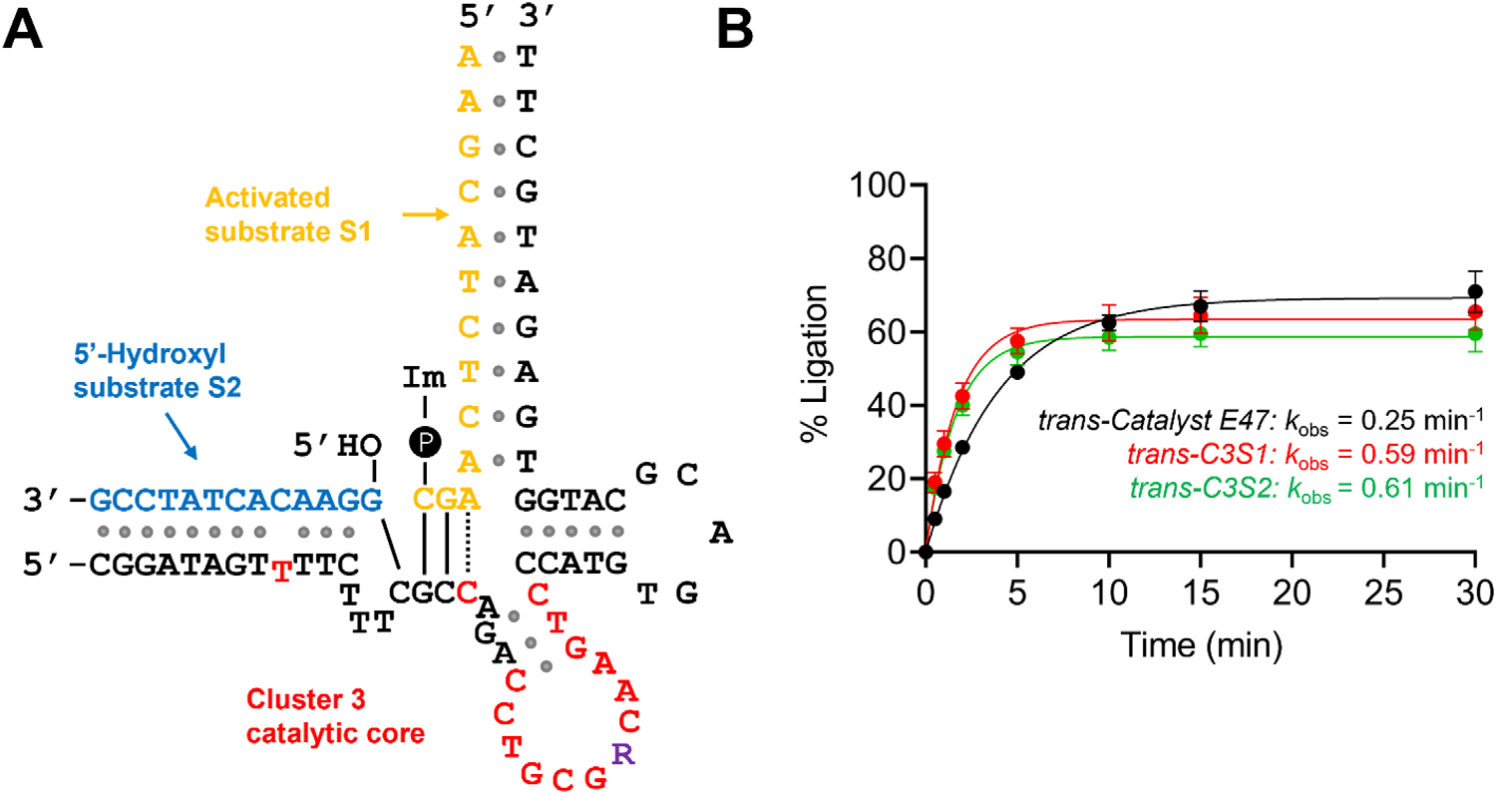
Proposed secondary structure of the consensus cluster 3 sequence in *trans* configuration (A). Unaltered nucleotides of cluster 3 compared to the original E47 sequence are shown in black, while altered nucleotides are shown in red, and **R** indicates a purine. Comparison of the DNA ligation kinetics of *trans*‐E47 (black line), *trans*‐C3S1 (red line), and *trans*‐C3S2 (green line), with the observed rate constant (*k*_obs_) generated using curve fitting to the one‐phase exponential association equation ($Y=Y_{max}\left(1-e^{-k_{obs}t}\right)$, performed with GraphPad Prism 8 (B).

Since cluster 3 showed the highest activity, C3S1 and C3S2 were selected for more detailed kinetic analysis. The rate of ligation was also evaluated for C6S1, which showed high activity, but further kinetics revealed that it was slower than E47, C3S1, and C3S2, so it was not investigated further (data not shown). In contrast to the *cis* versions of C3S1 and C3S2 used in the original activity screen, we performed kinetic assays in *trans* (Figure 3A) to better reflect their use in potential use in applications such as parallel ligation of data‐encoded sequences for DNA data storage [11].

Consistent with the initial screen, the fraction of S1 ligated by *trans*‐E47 after 60 min was slightly higher than by *trans*‐C3S1 and *trans*‐C3S2 (Figure 3B). This may be due to the larger sizes of C3S1 and C3S2 (60 nt) compared to E47 (47 nt), which provide more potential conformations, some of which may be inactive. Despite this, in‐depth kinetic analysis revealed that the rate of ligation by both *trans‐*C3S1 (*k*_obs_ = 0.59 min^−1^) and *trans*‐C3S2 (*k*_obs_ = 0.61 min^−1^) was over twofold faster than *trans*‐catalyst E47 (*k*_obs_ = 0.25 min^−1^) (Figure S5). Interestingly, C3S1 was 188‐fold more abundant than C3S2 in round 10 (3.2% vs 0.017%) despite showing near identical activity, which may indicate that the single nucleotide difference enhanced PCR amplification bias for C3S1.

The detailed kinetics also revealed an unexpected finding: the ligation rate of E47 in this study was fourfold higher than in the original report [20] (*k*_obs_ = 0.25 min^−1^ vs *k*_obs_ = 0.056 min^−1^), despite nearly identical reaction conditions. This difference may reflect less efficient phosphoimidazole activation of the DNA substrate in the original study, generating fewer viable substrates. The exact activation steps were not described in that study and were only referenced, so it was unknown how exactly the substrate was prepared [27].

Moreover, although our assays were performed under single‐turnover conditions, we hypothesize that all three *trans*‐acting DNAzymes may be capable of supporting multiple turnovers, based on prior observations that the original E47 DNAzyme can perform multiple turnovers in *trans* [20]. However, additional experimentation will be required to establish whether C3S1 and related variants can achieve efficient product release and sustained turnover. Nevertheless, single‐turnover conditions are likely sufficient for most applications, as DNAzymes can be readily supplied in excess of substrate.

### 2.3 Insights Into DNA Substrate Interactions by Cluster 3

**FIGURE 4:**
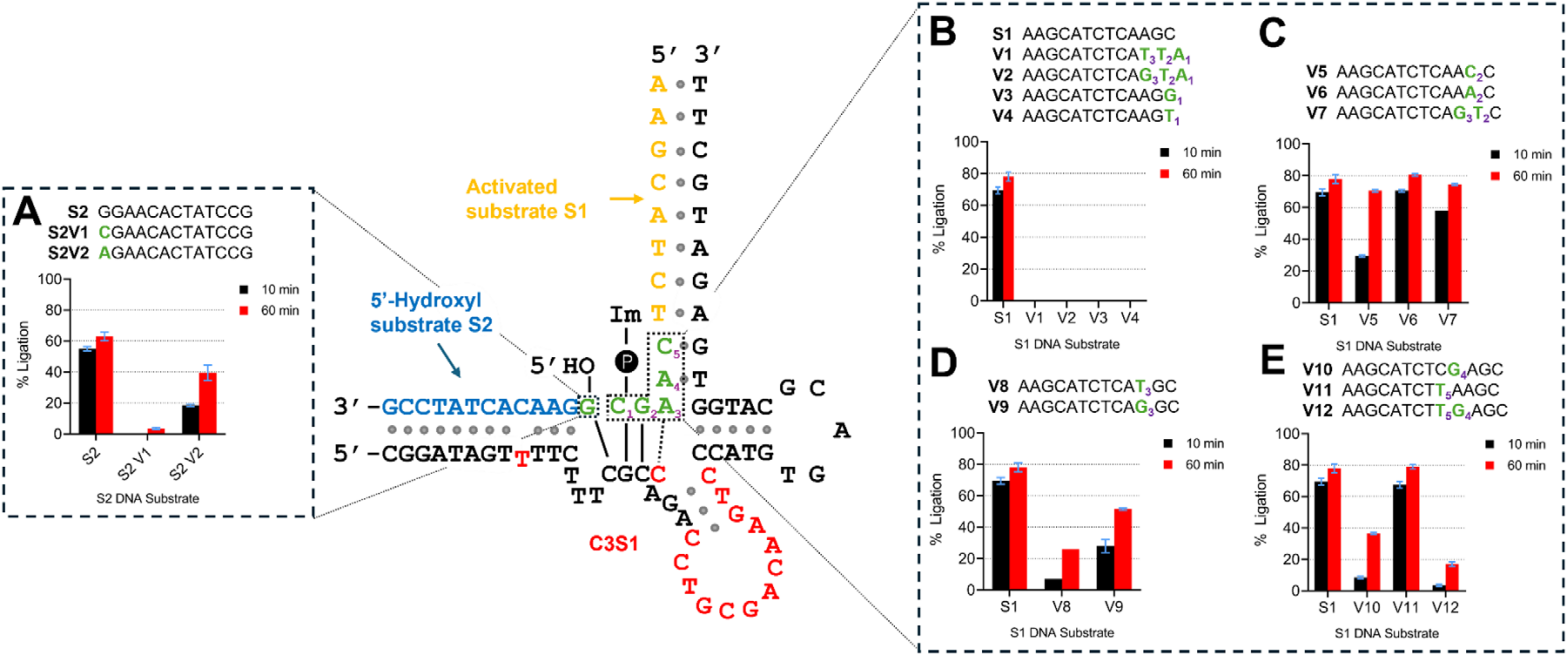
Substrate specificity requirements for C3S1 with S1 and S2. A total of 11 S1 DNA substrate variants were tested with *cis*‐C3S1, while two variants of the S2 DNA substrate were tested with *trans*‐C3S1. Sequence changes to the original S1 and S2 DNA substrates are shown in green. For S2, a 5′‐terminal G was required for *trans*‐C3S1 activity, with reductions in DNA ligation activity observed for both pyrimidine (S2V1) and purine (S2V2) nucleotides (A). For S1, V1‐V4 showed complete loss of *cis*‐C3S1 activity, indicating that 3′−terminal C at position 1 (C_1_) was vital for activity (B). G_2_ can be substituted for A_2_ without significant loss of activity, however reduction was observed when changed to C_2_ (C). *cis*‐C3S1 activity was reduced when A_3_ was changed to T_3_ or G_3_, showing a preference for a mismatch base pair with the catalytic core (D). Changing A_4_ to G_4_ caused a decrease in *cis*‐C3S1 activity through destabilizing the 3′ binding arm, while changing C_5_ to T_5_ had limited impact (E).

We performed detailed sequence analysis of cluster 3 using *cis*‐C3S1 with sequence‐modified S1 and S2 DNA substrates to elucidate the interaction network formed between C3S1 and the DNA substrates. We tested 12 different S1 variants and 2 different S2 variants and measured the ligation activity of *cis*‐C3S1 compared to unmodified versions of S1 and S2. For these studies, we performed all S1 DNA substrate activation and DNA ligation reactions at 16°C to improve stability of the phosphoimidazole intermediate, so that low‐yield ligations could be measured with better sensitivity. First, we tested if the 5′‐terminal G of S2 interacted with the catalytic core of C3S1 in a similar manner to the E47 DNAzyme. Changing to a pyrimidine (S2V1) or purine (S2V2) showed a significant loss in DNA ligation activity, with slightly higher activity observed in the latter, indicating the original G‐C interaction was favored (Figure 4A).

In a similar manner, we wanted to gain more insight into the sequence specificity of 3′‐terminal residues of S1 with C3S1. We designed two S1 variants, V1 and V2, with three altered 3′‐terminal nucleotides (C_1_G_2_A_3_) and observed a complete loss in DNA ligation activity (Figure 4B). We then tested if only the 3’‐terminal C_1_ of S1 was necessary for activity by changing it to a purine, G_1_ (V3), and a pyrimidine, T_1_ (V4), and again, observed complete loss in DNA ligation activity by C3S1, confirming that C_1_ is essential for activity, regardless of modification to adjacent nucleotides.

By contrast, G_2_ of the S1 DNA substrate was more tolerant to nucleotide substitution, with higher DNA ligation activity observed for purines (V5) compared to pyrimidines (V6) (Figure 4C). The identical DNA ligation activity observed for V6 compared to unmodified S1 points to a tolerable A_2_‐C mismatch between V6 and the C3S1 catalytic core. Interestingly, changing both G_2_ and A_3_ to T_2_ and G_3_ (V7) also resulted in relatively high DNA ligation activity by *cis*‐C3S1. This indicates that the C_2_‐C mismatch formed between V5 and the catalytic core can be stabilized by G_3_ when changed to another pyrimidine, such as T_2_ in this case.

Lower DNA ligation activity was observed when A_3_ of the S1 DNA substrate was changed to a pyrimidine, T_3_ (V8), or another purine, G_3_ (V9), with slightly higher DNA ligation activity observed with the latter (Figure 4D). Looking at the proposed secondary structure of C3S1, A_3_ appeared to form a mismatch pair with the 3′‐terminal C of the CGCC catalytic core motif, as lower DNA ligation activity was observed when A_3_ was changed to G_3_ (V9), forming a canonical WC base pair. The mutation of T→C in the CGCT motif of E47 compared to the CGCC motif of cluster 3 may be the key to the higher DNA ligation activity of C3S1 and C3S2, as mentioned in the previous section. The mismatch A_3_‐C pair between S1 and the CGCC motif of the cluster 3 catalytic core may provide improved flexibility of the catalytic core, facilitating better position of a catalytic Zn^2+^ or alignment of the 5′‐hydroxyl of S2 and 3′‐phosphoimidazole of S1 with nucleotides involved in catalysis, compared to the more rigid CGCT motif of E47. A similar phenomenon was observed in resolved structures of the well‐studied RNA‐cleaving 10–23 DNAzyme [28, 29], where an unbound pair between the RNA substrate and recognition arm of the 10–23 DNAzyme improved the conformational flexibility and activity of the catalytic core [30, 31].

C3S1 was significantly less tolerant to alteration of A_4_ (V10) of the S1 DNA substrate compared to C_5_ (V11), which showed nearly identical DNA ligation activity as unmodified S1 (Figure 4E). This was unsurprising, as A_4_ formed a terminal base pair with the 3′ binding arm of C3S1, making this position critical for overall binding stability with the S1 DNA substrate and positioning the 3′ phosphoimidazole of S1 proximal to the 5′‐hydroxyl of S2. Likewise, when A_4_ was altered in V12, a predictable reduction in DNA ligation activity was observed, despite an alteration in C_5_ as well.

The S1 and S2 variant analyses suggested that C3S1 tolerates several substrate mismatches near the ligation site; however, it remained unclear whether WC complementarity was required for activity. To address this, we generated C3S1 variants containing complementary nucleotides at positions that interact directly with the S1 and S2 substratevariants. Four compensatory C3S1 variants (S1V3_comp, S1V6_comp, S1V8_comp, and S2V1_comp) were tested with their corresponding substrates (Figure S6). S1V3_comp and S2V1_comp showed no detectable ligation, indicating that the native C_1_‐G (S1–C3S1) and G–C (S2–C3S1) pairs are essential and cannot be replaced by other WC pairs. S1V6_comp showed only limited activity, suggesting that both the original WC pair (G_2_‐C) and the A_2_‐C mismatch are preferred over the fully complementary A_2_–T pair. In contrast, S1V8_comp retained high activity, demonstrating that a T_3_‐A WC pair is tolerated, though the native A_3_‐C mismatch resulted in the highest ligation efficiency. Together, these findings suggest that C3S1 relies on a combination of specific WC pairs and catalytically beneficial mismatches at the ligation interface.

Finally, we decided to compare the substrate sequence specificity of *cis*‐C3S1 to the original *cis‐*E47 DNAzyme to see if greater substrate sequence versatility by C3S1 was generated in the selection process. We tested several S1 and S2 substrate variants with mutations at key positions. For S1V4 (position 1), S1V6 (position 2), S1V9 (position 3), and the 5′‐terminal position of S2 (S2V2), there was virtually no difference in DNA ligation activity between C3S1 and E47 (Figure S7). However, a significant difference in activity was observed with S1V11 (position 5), where C3S1 retained DNA ligation activity, while E47 was virtually inactive. This indicates that C3S1 can tolerate greater base mismatch within the S1 DNA substrate recognition arm compared to E47.

Overall, these analyses showed that the 5′‐terminal G of S2, as well as A_3_ and A_4_ of S1, can tolerate some degree of nucleotide substitution, though typically with reduced ligation efficiency. Alteration of C_1_ in S1 caused a complete loss of activity and remains essential for catalysis. In contrast, C3S1 tolerates substitutions at G_2_ and C_5_ of S1 without substantial loss in ligation efficiency. The A_3_ position is particularly interesting: although both T_3_ and G_3_ substitutions with WC pairing are tolerated, the original A_3_‐C mismatch consistently yielded the highest activity. These findings highlight that C3S1 employs a mixture of strict sequence requirements and beneficial mismatch interactions to support efficient ligation. Lastly, there is little difference in the substrate specificity between C3S1 and E47 for nucleotides that interact with the catalytic core, but the S1 DNA substrate recognition arm of C3S1 is more tolerant of mutation than E47. Therefore, for future applications, substrate sequences are limited to a 3′‐terminal G of S2 and C_1_ and A_4_ of S1, while nucleotide substitutions at other positions still yield active C3S1 DNAzymes.

### 2.4 Stabilization of the Activated S1 DNA Substrate

The original E47 DNAzyme required a DNA substrate with an “activated” terminal 3′‐phosphoimidazole moiety, which was generated by incubating the S1 DNA substrate with a carbodiimide, EDC, that covalently attached an imidazole to the 3′‐terminal phosphate group (Figure S8) [32, 33]. In addition to facilitating DNA ligation, EDC‐mediated activation of phosphates with imidazole is a critical step in a variety of biochemical processes involving nucleic acids, including modification [34], labeling [35], immobilization [36], and bioconjugation [37]. However, a significant problem with activated phosphoimidazole nucleic acids is that they are highly unstable and hydrolyze rapidly at room temperature, limiting their widespread utility. Several studies have increased the length of the activation reaction at lower temperatures to obtain high DNA ligation yields, but this is not feasible for applications at room temperature or higher [38, 39]. To improve the stability of the activated phosphoimidazole DNA substrate, we explored chemical alternatives to imidazole that would confer greater stability, while simultaneously enabling equal or greater DNA ligation activity, using *trans*‐C3S1 as a model DNA‐ligating DNAzyme.

**FIGURE 5:**
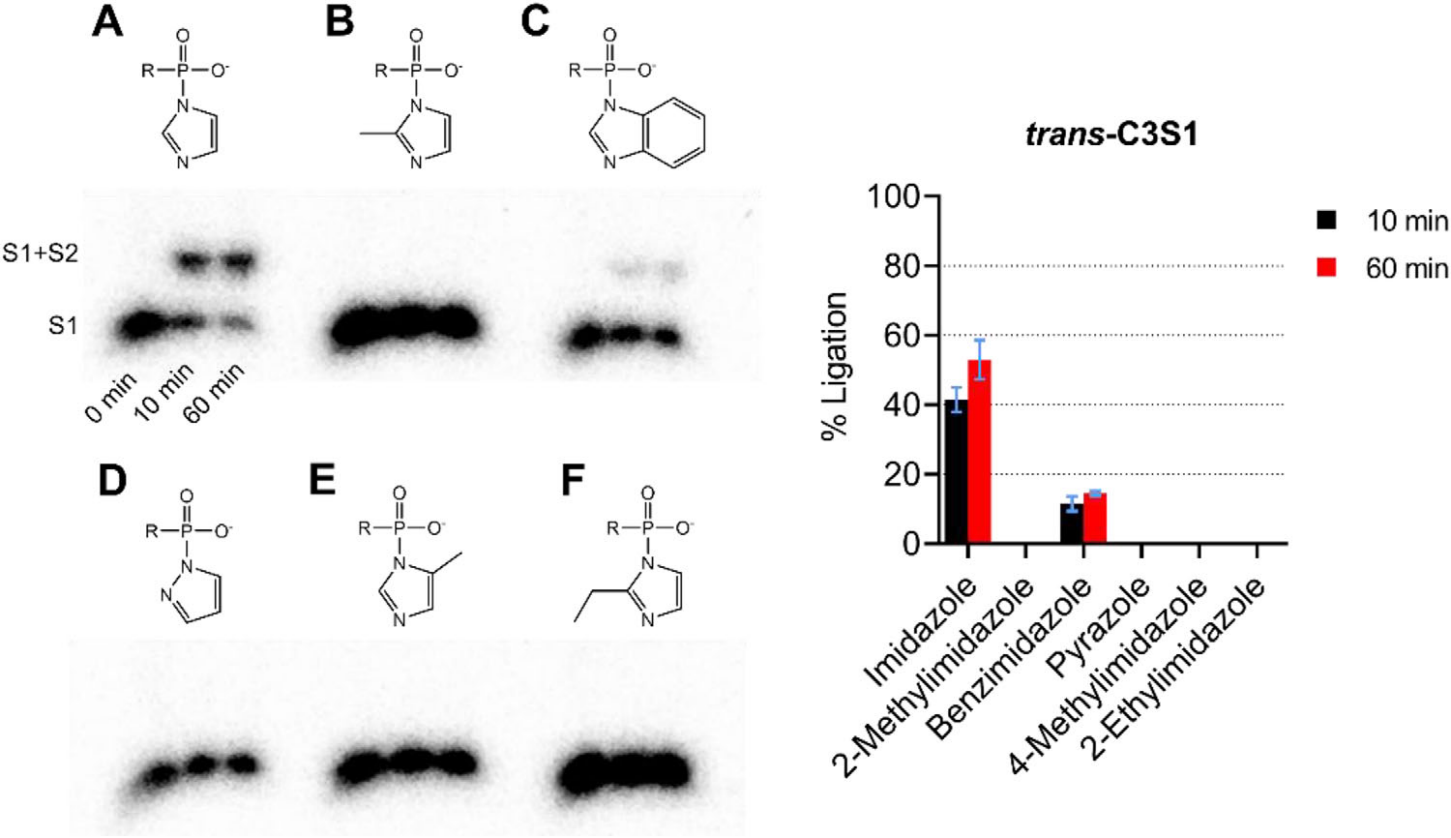
DNA ligation activity of *trans*‐C3S1 with different imidazole derivatives for activation of the S1 DNA substrate. The imidazolide compounds tested were imidazole (A), 2‐methylimidazole (B), benzimidazole (C), pyrazole (D), 4‐methylimidazole (E), and 2‐ethylimidazole (F). Activation reactions were conducted at room temperature (23°C) for 1 h and analyzed by 10% denaturing PAGE with 8 M urea.

We tested a total of five different imidazolide compounds that were found to promote nonenzymatic DNA ligation in a previous study [40]. The following compounds were substituted for imidazole (Figure 5A) in the S1 DNA substrate activation reaction for 1 h at room temperature: 2‐methylimidazole (Figure 5B), benzimidazole (Figure 5C), pyrazole (Figure 5D), 4‐methylimidazole (Figure 5E), and 2‐ethylimidazole (Figure 5F), and the DNA ligation efficiency of *trans*‐C3S1 was measured. Benzimidazole was the only imidazolide compound that yielded any detectable ligation products following activation of the S1 DNA substrate; however, the amount of S1+S2 ligation products was lower compared to the amount of S1+S2 produced by *trans*‐C3S1 with imidazole activation.

**FIGURE 6:**
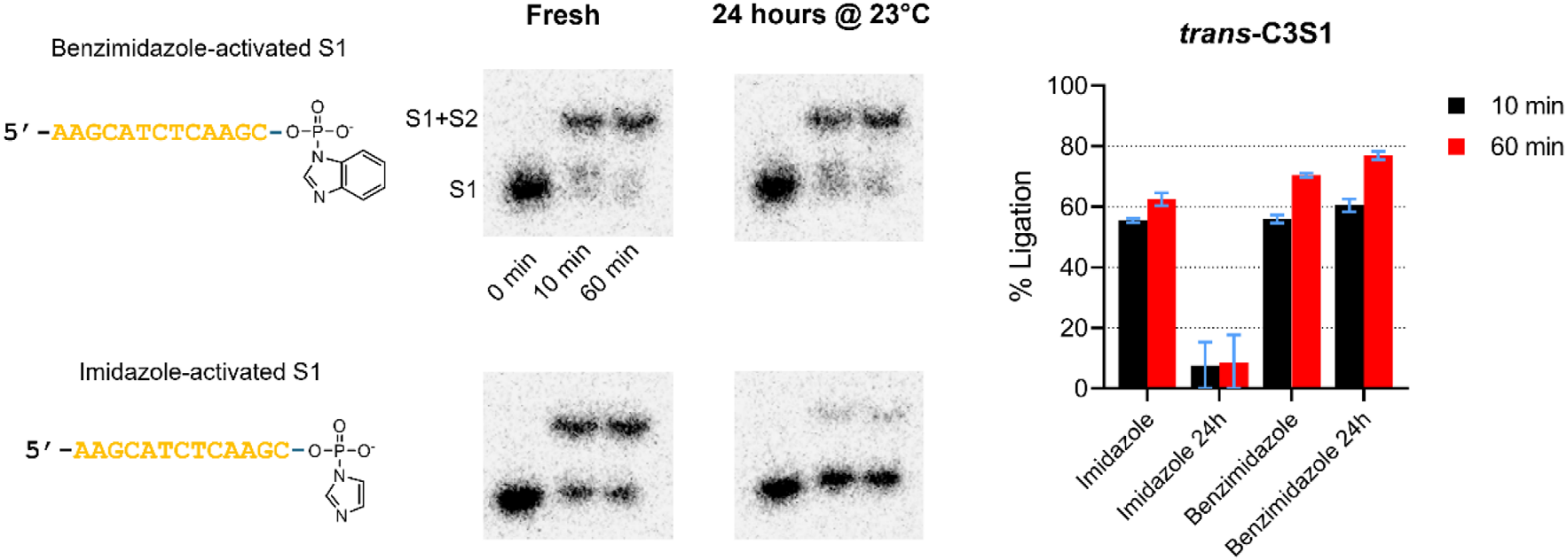
Stability of benzimidazole‐activated S1 DNA substrate compared to imidazole‐activated S1 at room temperature (23°C). Freshly purified benzimidazole‐ and imidazole‐activated S1 DNA substrates were incubated with *trans*‐C3S1 and S2 DNA substrates at room temperature, showing high activity with both substrates. After sitting at room temperature for 24 h, the purified benzimidazole‐ and imidazole‐activated S1 DNA substrates were again incubated with *trans*‐C3S1 and S2 at room temperature, showing that imidazole‐activated S1 was nearly completely degraded, while benzimidazole‐activated S1 was still stable.

Next, we tested whether the benzimidazole‐activated S1 DNA substrate was more stable at room temperature compared to the substrate activated with imidazole, and if ligation activity could be improved with benzimidazole. First, separate S1 DNA substrates were activated with imidazole and benzimidazole, then purified by removing unreacted imidazolides. While the S1 activation reaction with imidazole was incubated for 1 h at room temperature, the benzimidazole activation reaction was incubated for 24 h to improve the yield of activated phosphobenzimidazole S1 DNA substrate, since yields were significantly lower with a 1 h incubation time. A portion of the freshly purified S1 DNA substrates was then incubated with *trans*‐C3S1 at room temperature, and showed comparable DNA ligation efficiency (Figure 6). We note that variation in the ligation efficiency of imidazole‐activated S1 is observed between this experiment and the initial imidazolide screen. This is likely due to the high temperature sensitivity of the phosphoimidazole intermediate; even small fluctuations in ambient temperature during the year (typically 21–25°C in the laboratory) can lead to different levels of substrate degradation following purification. The remaining portion of the same purified imidazole‐ and benzimidazole‐activated S1 DNA substrates was left at room temperature for 24 h and then used as substrates for a subsequent DNA ligation reaction with *trans*‐C3S1. We observed that while imidazole‐activated S1 DNA substrate was degraded, as shown by a significant reduction in S1+S2 product formation by *trans*‐C3S1, the benzimidazole‐activated S1 DNA substrate was unaffected, and in fact showed slightly higher S1+S2 product formation.

While the benzimidazole‐activated S1 DNA substrate clearly shows lower susceptibility to temperature‐mediated hydrolysis compared to imidazole, the exact mechanism is unknown. However, we attribute the slight increase in DNA ligation activity after 24 h with benzimidazole‐activated S1 to slow conversion between structural isomers of benzimidazole, which may increase the proportion of viable phosphobenzimidazole isomers over time [41].

## 3 Conclusion

In summary, this study demonstrates that in vitro selection using a pre‐structured DNA library can generate DNAzymes with improved ligation activity, surpassing all previously reported DNA‐ligating DNAzymes. For applications where nonprotein ligation is preferred, sequence‐specific DNA ligation using DNAzymes offers a more stable and cost‐effective alternative. For example, several DNA‐based digital data storage strategies require the ligation of multiple DNA oligonucleotides to generate a readable sequence–a process typically facilitated by T4 DNA ligase [42, 43]. Although the E47 DNAzyme has been used as a more stable substitute, its slow ligation rate limits its practical application in this context [11, 44]. The use of *trans*‐acting DNAzymes such as C3S1 or C3S2, in combination with the highly stable phosphobenzimidazole‐based substrate activation chemistry, could theoretically double the rate of DNA code writing compared to E47, while also enhancing the long‐term stability of the DNA substrate.

Despite the progress made, there remains considerable room for improving the ligation efficiency of DNAzymes through in vitro selection. While the pre‐structured library approach enabled the discovery of faster DNA‐ligating DNAzymes, the structural diversity of the resulting sequences was limited, with many top variants still retaining elements of E47. Future in vitro selection efforts should consider starting with fully random DNA libraries and employ strategies that encourage exploration of broader sequence and structural space. This approach may lead to the identification of new DNAzyme architectures with even greater ligation activity.

## 4 Experimental Section

### 4.1 DNA Substrate Radiolabeling and Activation

All S1 DNA substrates were ordered from Integrated DNA Technologies (IDT), with sequences shown in Table S2, and 5′‐radiolabeled with ^32^P as follows: 16 nmol S1 DNA substrate was added to a solution containing ∼5 µCi γ‐^32^P ATP and 5 units of T4 polynucleotide kinase 3′ phosphatase minus (PNK‐3′P, New England Biolabs) in 10 µl of 1× PNK buffer (70 mM Tris‐HCl pH 7.6, 10 mM MgCl_2_, 5 mM DTT; New England Biolabs) and incubated for 30 min at 37°C. The reaction was stopped by the addition of 1× quenching buffer (10% sucrose, 0.5× TBE, 30 mM EDTA, 8 M urea, and 0.14 mg/mL bromophenol blue and xylene cyanol) and then purified by dPAGE containing 8 M urea. The gel was exposed on an Amersham Biosciences storage phosphor screen and imaged on an Amersham Typhoon 9200 scanner with a photo‐multiplier tube sensitivity of 4000 V. The 5′‐^32^P labeled S1 DNA substrates were then excised, crushed, soaked in 1× elution buffer (200 mM NaCl, 10 mM Tris–HCl pH 7.5, and 1 mM EDTA), and EtOH precipitated. The dried S1 DNA substrate pellets were then resuspended in 40 µl of dH_2_O with an approximate concentration of 200 µM.

The 3′ phosphate of all 5′‐^32^P labeled S1 DNA substrates was activated with imidazole prior to use in DNA ligation experiments. Activation reactions contained 600 pmol of 5′‐^32^P‐labeled S1 DNA substrate, 100 mM 1‐Ethyl‐3‐(3‐dimethylaminopropyl)carbodiimide (EDC)‐HCl, and 30 mM imidazole, with a final volume of 50 µl, and were incubated for 1 h at room temperature. Activations with other imidazolides, such as benzimidazole, were also performed at a concentration of 30 mM. The reaction was then purified using 0.5 mL Zeba spin desalting column (ThermoFisher Scientific) with a 7K molecular weight cutoff, prepared according to manufacturer instructions. Following the addition of the activation reaction, the column was spun for 2 min at 1500 rpm. The purified, imidazole‐activated, 5′‐^32^P‐labeled S1 DNA substrate, with an approximate concentration of 6 µM, was immediately used in DNA ligation reactions.

### 4.2 In Vitro Selection

All sequences used for in vitro selection were ordered from IDT, with larger sequences (>25 nt) purified by dPAGE containing 8 M urea, followed by EtOH precipitation. Each round of in vitro selection contained ∼50 pmol of purified, imidazole‐activated, 5′‐^32^P‐labeled S1 DNA substrate prepared as above and *cis*‐E47 pre‐structured library. Reactions were initiated by the addition of 1x reaction buffer (4 mM ZnCl_2_, 400 mM NaCl, and 30 mM HEPES, pH 7.0) and halted by the addition of 1x quenching buffer following reaction completion. Library amounts and reaction times for each round of in vitro selection are shown in Figure S1. The reaction mixture was subjected to dPAGE, exposed on a storage phosphor screen, and imaged as described previously. The DNA ligation products were then excised from the gel, crushed, soaked in 1 × elution buffer, and EtOH precipitated.

The DNA pellet was then resuspended in 50 µl of water, where 1 µl was used as DNA template for qPCR1, which contained 500 nM forward and reverse primers, 200 µM dNTPs, 2.5U Taq DNA polymerase (Thermo Fisher Scientific), 1x Taq DNA polymerase buffer (Thermo Fisher Scientific), and 1x EvaGreen dye (Biotium), and was run until the reaction plateaued. The qPCR1 amplification product was diluted 1:10 with water, where 1 µl was used as a template for qPCR2 with a total of 30 reactions. The reaction components for qPCR2 were identical to qPCR1, except that the 500 nM reverse primer was substituted with 500 nM blocked reverse primer, and the reaction was run until the reaction plateaued. The eluent reactions were pooled, and EtOH precipitated, followed by dPAGE purification, where the sense strand was excised, crushed, soaked in 1× elution buffer, and EtOH precipitated again. The DNA was resuspended in H_2_O and quantified with a NanoVue Plus Spectrophotometer (VWR) and used for the subsequent round of in vitro selection.

### 4.3 DNA Ligase DNAzyme Kinetic Reactions

All DNA ligase DNAzyme kinetic reactions were performed in triplicate at room temperature under single‐turnover conditions. Reactions contained 1 µM DNAzyme, ∼0.5 µM purified, imidazole‐activated, 5′‐^32^P‐labeled S1 DNA substrate, and 1x reaction buffer. For *trans* DNAzyme kinetic reactions, 1 µM S2 DNA substrate was also added. For simple DNAzyme kinetic reactions, timepoints were taken at 10 and 60 min, where an aliquot of the reaction was added to 1x quenching buffer. For in‐depth DNAzyme kinetic reactions, timepoints were taken at 0.5, 1, 2, 5, 10, 15, 30, and 60 min and quenched as described previously. Each timepoint was analyzed by dPAGE, exposed on a storage phosphor screen, and imaged on an Amersham Typhoon. The ligation bands were quantified using ImageJ software by measuring the intensity ratio between ligated S1 DNA substrate and total (ligated + nonligated) S1 DNA substrate for each reaction. In‐depth DNAzyme kinetic reactions were fit to the one‐phase exponential association equation $Y=Y_{max}\left(1-e^{-k_{obs}t}\right)$, using GraphPad Prism 8, to obtain the first‐order rate constant, *k*_obs_.

## Author Contributions

**Connor Nurmi**: data collection, formal analysis, writing – original draft. **Yingfu Li**: conceptualization, writing – review & editing. **Gemma Mendonsa**: conceptualization, writing – review & editing. **Mengdi Bao**: writing – review & editing.

## Conflicts of Interest

The authors declare no conflict of interest.

## Supporting information




**Supporting File 1**: chem70527‐sup‐0001‐SuppMat.pdf

## Data Availability

The data that support the findings of this study are available in the supplementary material of this article.
