# Supplementary material for: Evolved DNAzymes and Stable Activation Chemistry Enable High‐Efficiency DNA Ligation
Source: Chemistry. 2025 Dec 5;32(6):e02788. doi: 10.1002/chem.202502788 (PMC12887632; doi:10.1002/chem.202502788)
Supplement: Supplementary file 1 — Supporting File 1: chem70527‐sup‐0001‐SuppMat.pdf [file CHEM-32-e02788-s001.pdf]

Evolved DNAzymes and Stable Activation Chemistry Enable High-Efficiency DNA Ligation

Connor Nurmi, Gemma Mendonsa, Mengdi Bao and Yingfu Li

Supplementary Information

Table S1. Comparison of reported DNAzymes with DNA ligation activity.

DNAzyme	Rate	Configuration	Notes	Reference
L115	0.001 min^{-1}	*trans*	Limited substrate sequence versatility	[1]
C1	0.033 min^{-1}	*cis*	Requires chemically modified substrates	[2]
8LV13	0.1 min^{-1}	*trans*	Forms non-canonical 5'-2' linkages	[3]
E47	0.056 min^{-1}	*trans*	Reduced sequence versatility of terminal substrate sequences	[4]
E47	0.25 min^{-1}	*trans*	Reduced sequence versatility of terminal substrate sequences	This study
C3S1	0.59 min^{-1}	*trans*	Reduced sequence versatility of terminal substrate sequences	This study
C3S2	0.61 min^{-1}	*trans*	Reduced sequence versatility of terminal substrate sequences	This study

Table S2. List of all sequences used in this study.

**Name**	**Sequence (5′→3′)**	**Length**
cis-E47 pre-structured library	GGAACACTATCCGTTTGACGGATAGTGTTC**NNNNNNNNNN**CGCT**NNNNNNNNNN**CCATGTGACGCATGGTGAGATGCTT	79
Forward primer	GGAACACTATCCGTTTGACGGA	22
Reverse primer	AAGCATCTCACCATGCGTCACA	22
Blocked reverse primer	TTTTTTTTTTTTTTTTTTTT/iSP18/AAGCATCTCACCATGCGTCACA	42
cis-E47	GGAACACTATCCGTTTGACGGATAGTGTTCTTTCGCTAGACCATGTGACGCATGGTGAGATGCTT	65
cis-C1S1	GGAACACTATCCGTTTGACGGATAGTGTTCGTTGTTCGTTCGCTAGACCTGAGGCCATGTGACGCATGGTGAGATGCTT	79
cis-C1S2	GGAACACTATCCGTTTGACGGATAGTGTTCGGTGTTCGTTCGCTAGACCTGAGGCCATGTGACGCATGGTGAGATGCTT	79
cis-C1S3	GGAACACTATCCGTTTGACGGATAGTGTTCGGTGTTCGTTCGCTAGACCTGTGGCCATGTGACGCATGGTGAGATGCTT	79
cis-C1S4	GGAACACTATCCGTTTGACGGATATTGTTCGGTGTTCGTTCGCTAGACCTGTGTCCATGTGACGCATGGTGAGATGCTT	79
cis-C2S1	GGAACACTATCCGTTTGACGGATAGTGTTCGTTTCGGTGGCGCTATTTACGGGTCCATGTGACGCATGGTGAGATGCTT	79
cis-C2S2	GGAACACTATCCGTTTGACGGATAGTATTCGTTTCGGTGGCGCTATTTACGGGTCCATGTGACGCATGGTGAGATGCTT	79
cis-C3S1	GGAACACTATCCGTTTGACGGATAGTTTTCTTTCGCCAGACCTGCGACAAGTCCCATGTGACGCATGGTGAGATGCTT	78
cis-C3S2	GGAACACTATCCGTTTGACGGATAGTTTTCTTTCGCCAGACCTGCGGCAAGTCCCATGTGACGCATGGTGAGATGCTT	78
cis-C4S1	GGAACACTATCCGTTTGACGGATAGTATTCTGTGATCTTTCGCTAGACCAAAGTCCATGTGACGCATGGTGAGATGCTT	79
cis-C5S1	GGAACACTATCCGTTTGACGGATAGTATTCGAGTGTCGTTCGCTAGACCTGAGGCCATGTGACGCATGGTGAGATGCTT	79
cis-C6S1	GGAACACTATCCGTTTGACGGATAGTACTCGTTGTTCGTTCGCTAGACCAAAGGCCATGTGACGCATGGTGAGATGCTT	79
trans-E47	CGGATAGTGTTCTTTCGCTAGACCATGTGACGCATGGTGAGATGCTT	47
trans-C3S1	CGGATAGTTTTCTTTCGCCAGACCTGCGACAAGTCCCATGTGACGCATGGTGAGATGCTT	60
trans-C3S2	CGGATAGTTTTCTTTCGCCAGACCTGCGGCAAGTCCCATGTGACGCATGGTGAGATGCTT	60
S1 DNA substrate	AAGCATCTCAAGC-PO_4^{2-}	13
S1V1	AAGCATCTCATTA-PO_4^{2-}	13
S1V2	AAGCATCTCAGTA-PO_4^{2-}	13
S1V3	AAGCATCTCAAGG-PO_4^{2-}	13
S1V4	AAGCATCTCAAGT-PO_4^{2-}	13

S1V5	AAGCATCTCAACC-PO_4^{2-}	13
S1V6	AAGCATCTCAAAC-PO_4^{2-}	13
S1V7	AAGCATCTCAGTC-PO_4^{2-}	13
S1V8	AAGCATCTCATGC-PO_4^{2-}	13
S1V9	AAGCATCTCAGGC-PO_4^{2-}	13
S1V10	AAGCATCTCGAGC-PO_4^{2-}	13
S1V11	AAGCATCTTAAGC-PO_4^{2-}	13
S1V12	AAGCATCTTGAGC-PO_4^{2-}	13
S2 DNA substrate	GGAACACTATCCG	13
S2V1	CGAACACTATCCG	13
S2V2	AGAACACTATCCG	13

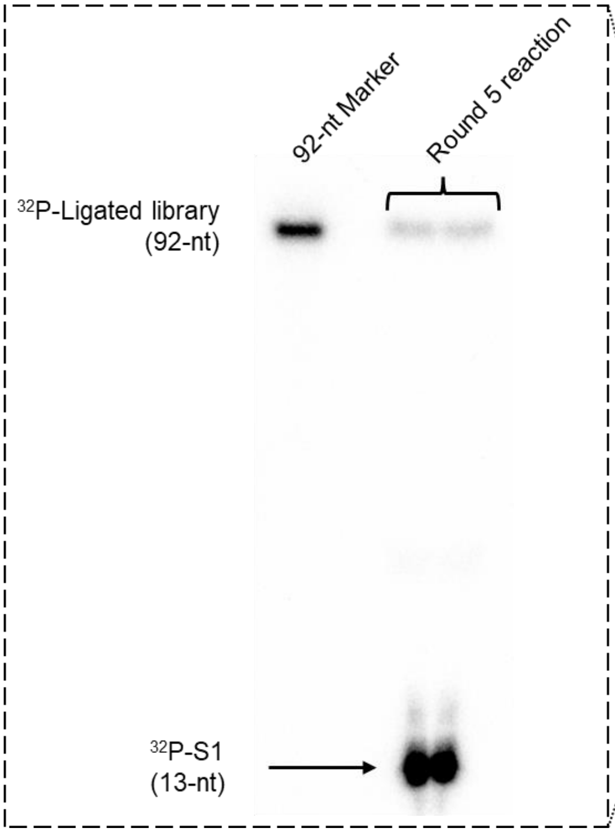

Round	Library amount	Incubation time	%Ligation
**1**	50 pmol	48 hr	0.36
**2**	25 pmol	18 hr	0.46
**3**	25 pmol	5 hr	0.51
**4**	25 pmol	2 hr	0.12
**5**	25 pmol	2 hr	7.4
**6**	25 pmol	30 min	7.5
**7**	25 pmol	5 min	6.6
**8**	25 pmol	30 s	2.0
**9**	25 pmol	30 s	2.2
**10**	10 pmol	30 s	1.1

Figure S1. Overview of dPAGE analysis of a round of *in vitro* selection using round 5 as an example (Left). Successfully ligated library sequences align with the 92-nt marker and were excised, amplified and used in the subsequent round of selection. Summary of experimental conditions used for each round of *in vitro* selection (Right). All reactions were conducted at room temperature in buffer containing 4 mM Zn^{2+}.

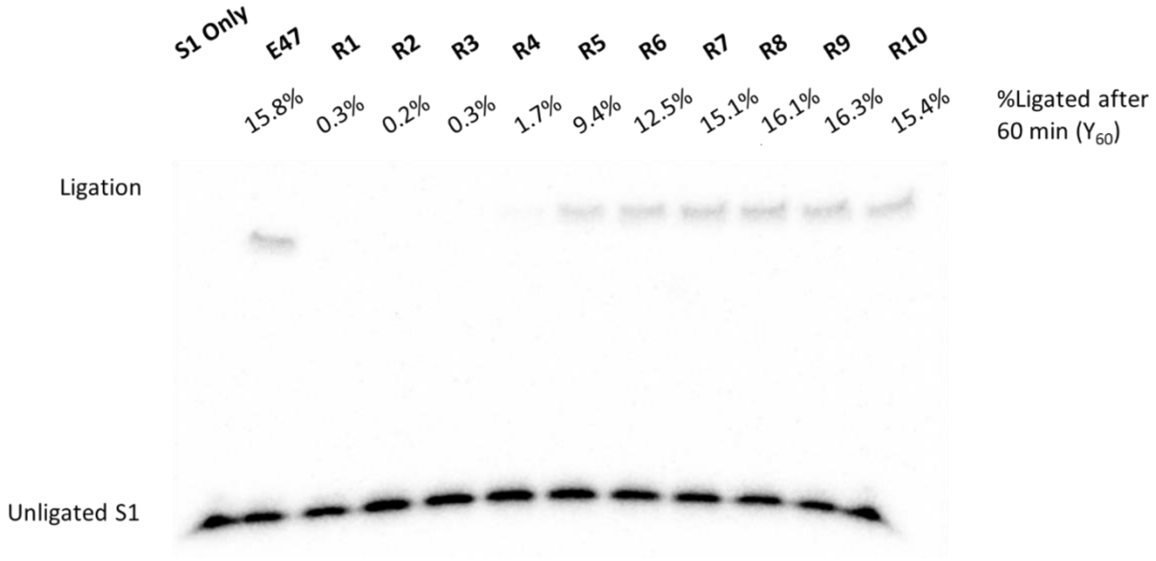

Figure S2. Single 60 min timepoint (Y_{60}) analysis of DNA ligation activity of the pool for each round of *in vitro* selection via dPAGE. Reactions were conducted at room temperature in buffer containing 4 mM Zn^{2+}. The ligation bands were quantified using ImageJ software by measuring the intensity ratio between ligated S1 DNA substrate and total (ligated + non-ligated) S1 DNA substrate for each reaction.

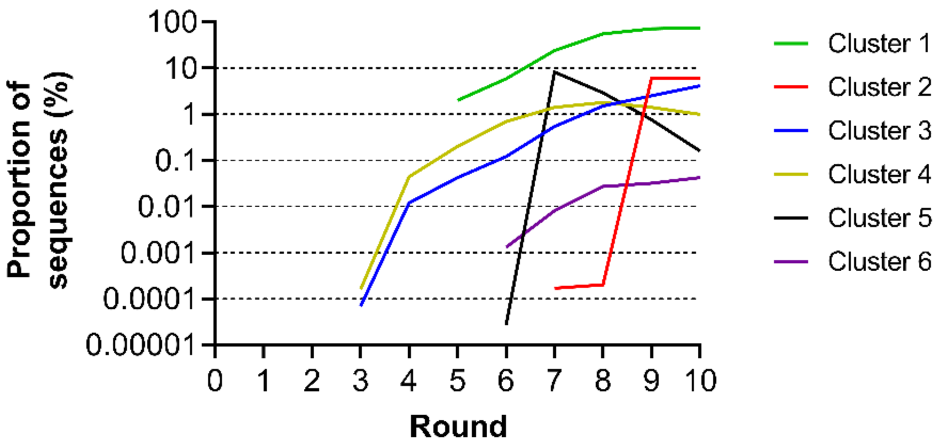

Figure S3. The trajectory of sequence clusters through each round of *in vitro* selection as a proportion of all sequence clusters in the pool.

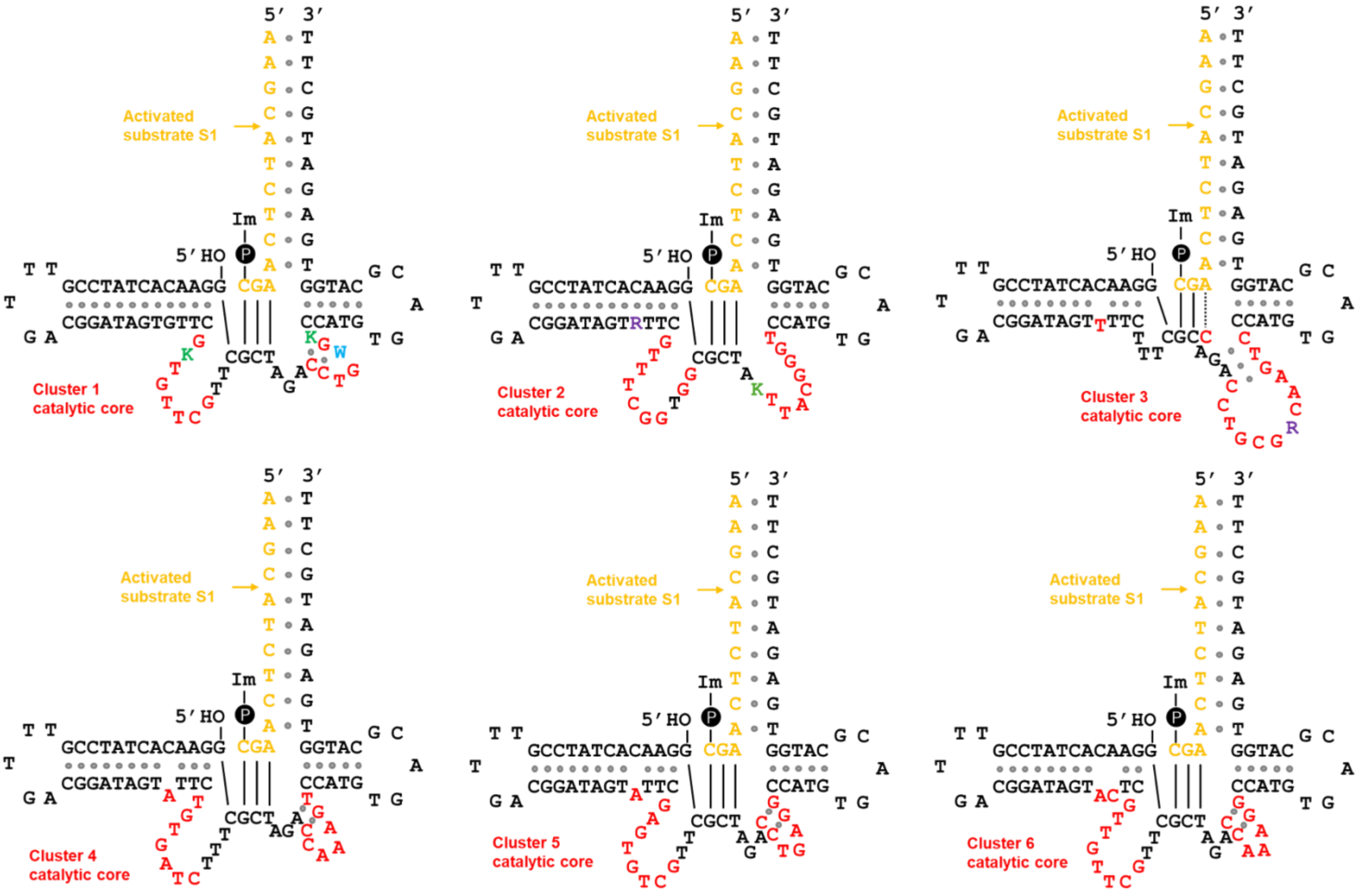

Figure S4. Secondary structures of the top six clusters chosen from the *in vitro* selection experiments. Yellow nucleotides reflect the activated S1 DNA substrate, while red nucleotides reflect changes in the catalytic core sequence compared to the original catalyst E47. **R** denotes purines, **W** denotes A or T and **K** denotes G or T.

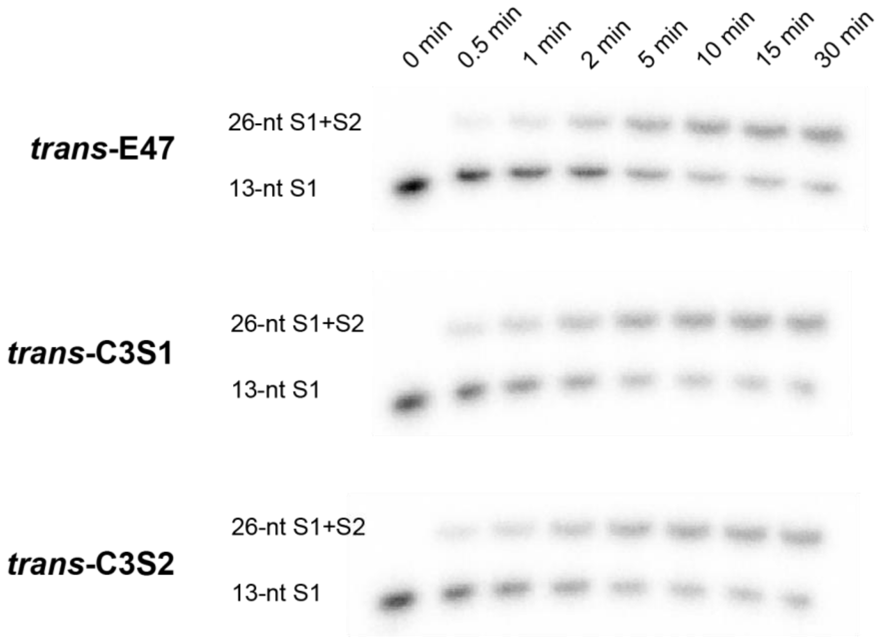

Figure S5. Denaturing PAGE containing 8 M urea used to determine the ligation kinetics of *trans*-acting cluster 3 sequences C3S1 and C3S2 compared to catalyst E47. Reactions were conducted at room temperature and initiated by addition of buffer containing 4 mM Zn^{2+}. The ligation bands were quantified using ImageJ software by measuring the intensity ratio between ligated S1 DNA substrate and total (ligated + non-ligated) S1 DNA substrate for each reaction.

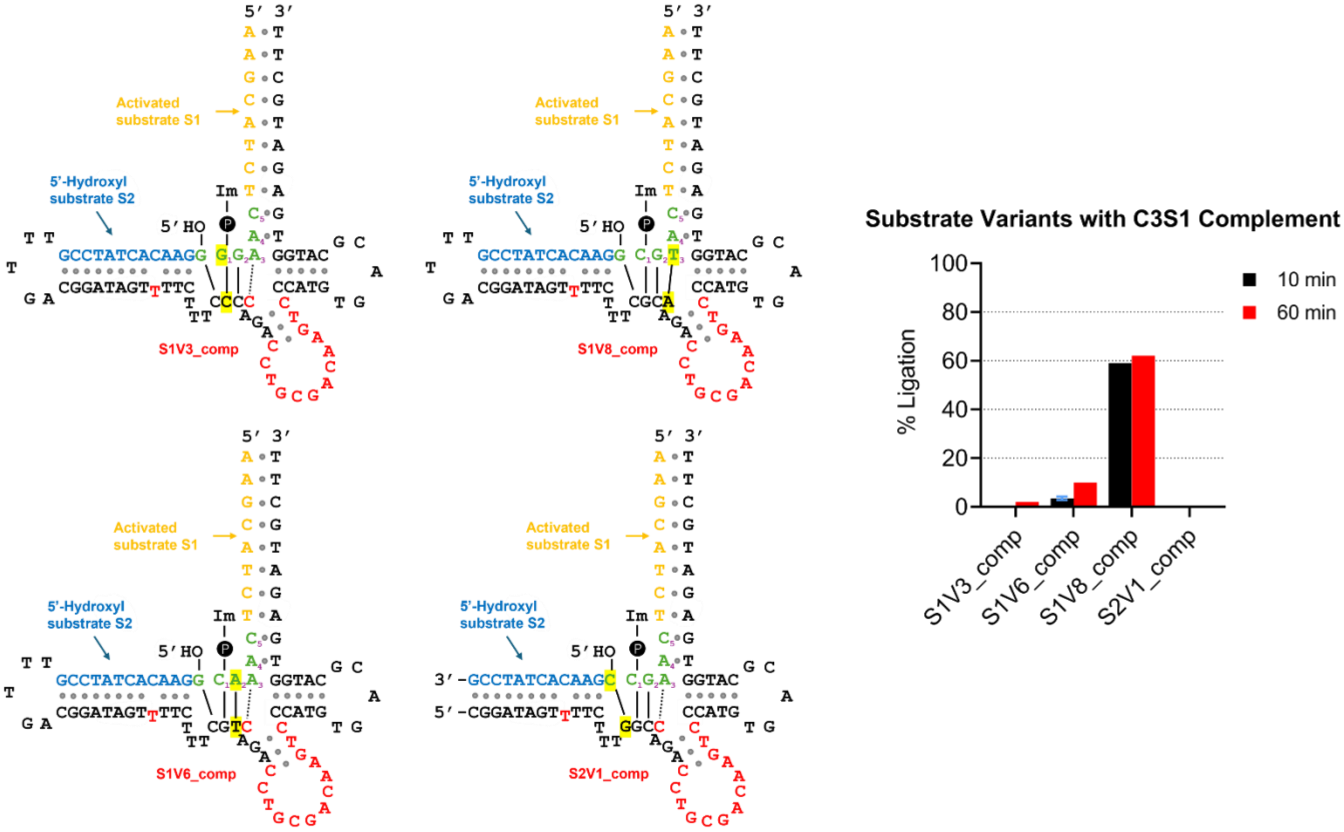

Figure S6. Analysis of Watson-Crick (WC) complementarity of C3S1 between C3S1 and S1/S2 DNA substrate variants. Ligation assays were performed using S1 and S2 substrate variants paired with C3S1 variants containing compensatory mutations (S1V3_comp, S1V6_comp, S1V8_comp, and S2V1_comp). Both S1V3 and S2V1 showed little to no activity with their respective complementary C3S1 variants, indicating that the original base pairs at these positions (C_1–G for S1 and G–C for S2) are required for ligation. S1V6 exhibited only minimal activity with S1V6_comp, suggesting a preference for the native G_2–C pair or the A_2–C mismatch over the fully complementary A_2–T pair. In contrast, S1V8 retained robust ligation activity with S1V8_comp, demonstrating that a T_3–A WC pair can be accommodated at this position, although the original A_3–C mismatch still supported the highest ligation efficiency.

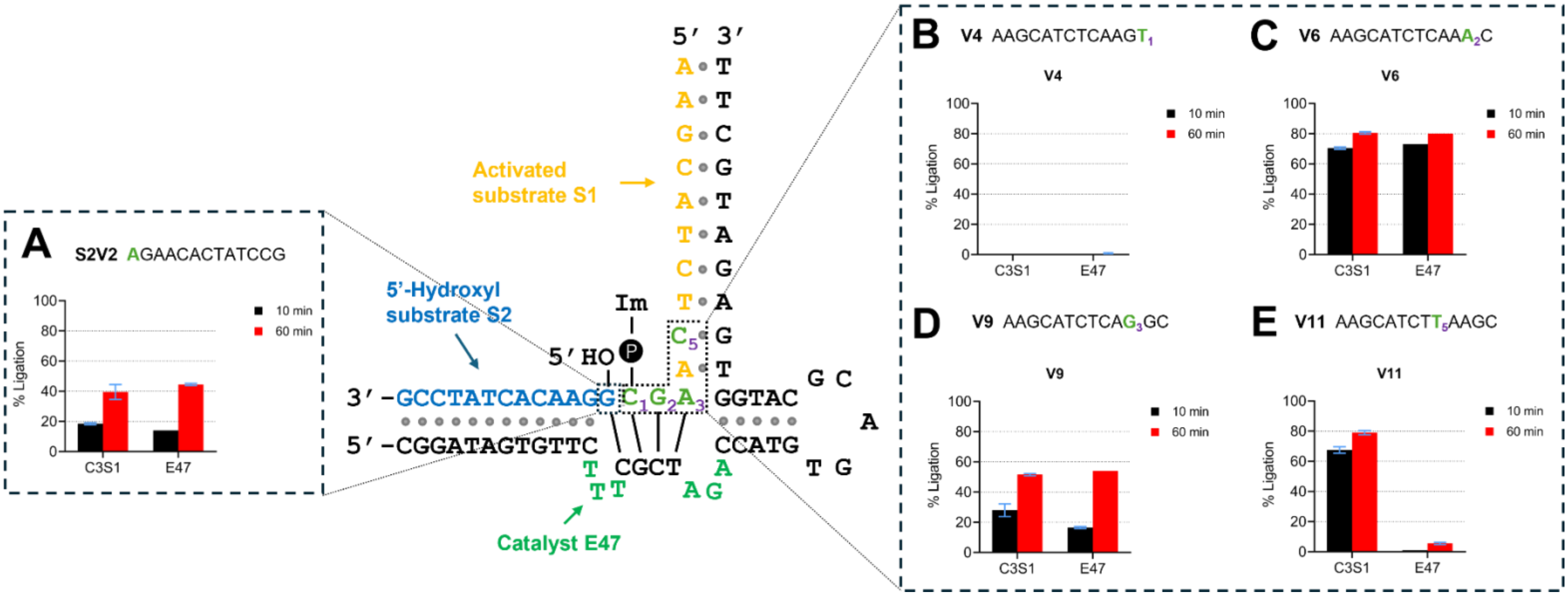

Figure S7. Substrate specificity comparison between E47 and C3S1 with S1 and S2. DNA ligation activity between E47 and C3S1 is comparable with S2V2 (**A**). The three nucleotides of S1 that interact with the catalytic core, $C_1G_2A_3$, also show comparable activity between E47 and C3S1 when mutated in S1V4 (**B**), S1V6 (**C**) and S1V9 (**D**). E47 is not tolerant to mutation at C_5 with S1V11 (**E**), while C3S1 retains full DNA ligation activity.

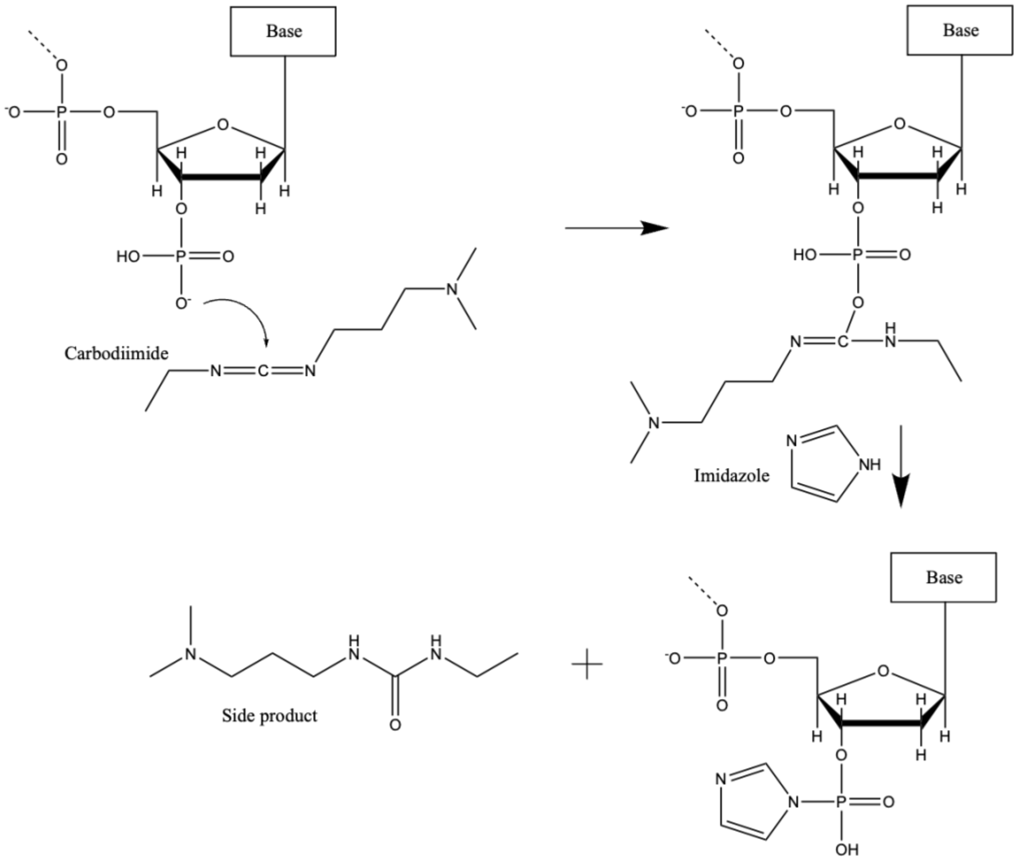

Figure S8. Mechanism of Carbodiimide (EDC-HCl)-mediated generation of phosphoimidazole DNA adopted from.[5]

References

[1] A. Sreedhara, Y. Li, R. R. Breaker, *J. Am. Chem. Soc.* **2004**, *126*, 3454–3460.
[2] M. Levy, A. D. Ellington, *Bioorg. Med. Chem.* **2001**, *9*, 2581–2587.
[3] C. S. Lee, T. P. Mui, S. K. Silverman, *Nucleic Acids Res.* **2011**, *39*, 269–279.
[4] B. Cuenoud, J. W. Szostak, *Nature* **1995**, *375*, 611–614.
[5] M. P. Wickramathilaka, B. Y. Tao, *J. Biol. Eng.* **2019**, *13*, 63.
